# Tattoos as a risk factor for malignant lymphoma: a population-based case–control study

**DOI:** 10.1016/j.eclinm.2024.102649

**Published:** 2024-05-21

**Authors:** Christel Nielsen, Mats Jerkeman, Anna Saxne Jöud

**Affiliations:** a Division of Occupational and Environmental Medicine, Department of Laboratory Medicine, Lund University, Lund, Sweden; b Division of Oncology, Department of Clinical Sciences Lund, Lund University, Lund, Sweden; c Department of Oncology, Skåne University Hospital, Lund, Sweden; d Division of Orthopaedics, Department of Clinical Sciences Lund, Lund University, Lund, Sweden

**Keywords:** Cancer, Lymphoma, Lifestyle-related risk factor, Cancer prevention, Population-based research

## Abstract

**Background:**

The popularity of tattoos has increased dramatically over the last few decades. Tattoo ink often contains carcinogenic chemicals, e.g., primary aromatic amines, polycyclic aromatic hydrocarbons, and metals. The tattooing process invokes an immunologic response that causes translocation of tattoo ink from the injection site. Deposition of tattoo pigment in lymph nodes has been confirmed but the long-term health effects remain unexplored. We used Swedish National Authority Registers with full population coverage to investigate the association between tattoo exposure and overall malignant lymphoma as well as lymphoma subtypes.

**Methods:**

We performed a case–control study where we identified all incident cases of malignant lymphoma diagnosed between 2007 and 2017 in individuals aged 20–60 years in the Swedish National Cancer Register. Three random age- and sex-matched controls per case were sampled from the Total Population Register using incidence density sampling. We assessed exposure through a questionnaire in 2021, and data on potential confounders were retrieved from registers. We used multivariable logistic regression to estimate the incidence rate ratio (IRR) of malignant lymphoma in tattooed individuals.

**Findings:**

The study population consisted of 11,905 individuals, and the response rate was 54% among cases (*n* = 1398) and 47% among controls (*n* = 4193). The tattoo prevalence was 21% among cases and 18% among controls. Tattooed individuals had a higher adjusted risk of overall lymphoma (IRR = 1.21; 95% CI 0.99–1.48). The risk of lymphoma was highest in individuals with less than two years between their first tattoo and the index year (IRR = 1.81; 95% CI 1.03–3.20). The risk decreased with intermediate exposure duration (three to ten years) but increased again in individuals who received their first tattoo ≥11 years before the index year (IRR = 1.19; 95% CI 0.94–1.50). We found no evidence of increasing risk with a larger area of total tattooed body surface. The risk associated with tattoo exposure seemed to be highest for diffuse large B-cell lymphoma (IRR 1.30; 95% CI 0.99–1.71) and follicular lymphoma (IRR 1.29; 95% CI 0.92–1.82).

**Interpretation:**

Our findings suggested that tattoo exposure was associated with an increased risk of malignant lymphoma. More epidemiologic research is urgently needed to establish causality.

**Funding:**

The Swedish Research Council for Health, Working Life and Welfare.

**Research in context**

**Evidence before this study**

We searched PubMed with the search terms lymphoma (all fields), and tattoo∗ (all fields), for publications from database inception to October 20, 2023. We identified only one study that addressed tattoos as a risk factor for lymphoma, but it was underpowered because of the small number of tattooed participants.

**Added value of this study**

To our knowledge, this is the first epidemiologic study to investigate the association between tattoo exposure and overall malignant lymphoma as well as lymphoma subtypes using a population-based case–control design and a large sample size. The study included all incident cases of malignant lymphoma that were diagnosed in individuals aged 20–60 years between 2007 and 2017 in Sweden, with three sex-matched controls per case. We found that tattooed individuals had a 21% higher risk of overall malignant lymphoma compared with non-tattooed individuals, and that the association was strongest for diffuse large B-cell lymphoma and follicular lymphoma.

**Implications of all the available evidence**

The study suggests that tattoos may be a risk factor for malignant lymphoma that is actionable from a public health perspective. Further epidemiologic research is needed to establish causality. The study underscores the importance of regulatory measures to control the chemical composition of tattoo ink.

## Introduction

The popularity of tattoos has increased dramatically over the last few decades. Today, several European studies report a prevalence above 20%,^1^, ^2^, ^3^ and the U.S. prevalence is estimated at approximately 30%.^4^ Most people get their first tattoo at a young age,^2^^,^^5^ which implies exposure to some chemical constituents of tattoo ink over almost the entire life course. Yet, research has only begun to scratch the surface of understanding the long-term health effects of tattoos.

Tattoo inks are cocktails of organic and inorganic colour pigments, together with precursors and byproducts from the pigment synthesis, and additives. Coloured inks may contain primary aromatic amines (PAA), black inks often contain polycyclic aromatic hydrocarbons (PAH), and metals (i.e., arsenic, chromium, cobalt, lead, and nickel) are found in ink of all colours.^6^^,^^7^ A significant and concerning number of chemicals in tattoo ink are classified as carcinogenic by the International Agency for Research on Cancer.^8^, ^9^, ^10^, ^11^

During the tattooing process, ink is injected into the dermis through repeated punctures of the skin barrier. When any antigen breaches the skin barrier, the local immunologic response includes cell-mediated translocation of the antigen to the local lymph nodes from where a systemic immune response is initiated. The translocation of tattoo ink seems to be very effective; it has been estimated that 32% of the injected pigment is translocated after 6 weeks,^12^ and that as much as 99% may become translocated over time.^13^

In clinical settings, pigmented and enlarged lymph nodes have been described in tattooed individuals for decades. Translocation of both black and coloured tattoo pigments to human lymph nodes has been confirmed,^14^^,^^15^ as have depositions of metal particles from tattoo needle wear.^16^ Lymph nodes contain proliferating cells and are sensitive targets for carcinogenic chemicals. There is mounting evidence that immunologic disruption from exposure to solvents, flame retardants, pesticides, and hair dyes plays a key role in the pathogenesis of malignant lymphoma.^17^

There has been a global rise in the incidence of malignant lymphoma that remains largely unexplained,^18^^,^^19^ and it is urgent to understand any association with parallel trends in lifestyle-related factors. To our knowledge, only one study has addressed tattoos as a risk factor for lymphoma. Warner et al. found no evidence of an increased risk of non-Hodgkin lymphoma in tattooed individuals.^20^ Their study was based on data collected between 2000 and 2004, which is quite early in the mainstreaming of tattoos, and the analysis was likely underpowered because of the small number of tattooed participants.

We aimed to investigate if tattoo exposure increases the risk of malignant lymphoma in a population-based case–control study leveraging Swedish National Authority Registers. In addition, we investigated exposure-response relationships and the effect of exposure duration by accounting for the time between the first tattoo and the index year. Finally, we explored the association between tattoo exposure and lymphoma subtypes.

## Methods

### Study design

We performed a population-based case–control study, nested within the total Swedish population. Cases were all individuals with incident malignant lymphoma, according to the International Classification of Diseases for Oncology, 3rd Edition (Table 1), diagnosed between 2007 and 2017 in Sweden.

**Table 1:** Included subtypes of malignant lymphoma and their aggregation into broader diagnostic groups.

Lymphoma subgroup^a^	Subtype	ICD-O^b^/3.2	ICD-O-3 morphology code	ICD-O/2	ICD-O-2 morphology code
*Hodgkin*	Lymphocyte-rich classic Hodgkin lymphoma	site	9651/3	C81.0	9658/3
	Nodular lymphocyte predominant Hodgkin lymphoma	site	9659/3	C81.0	9659/3
	Nodular sclerosis classic Hodgkin lymphoma	site	9663/3	C81.1	9663/3
	Mixed cellularity classic Hodgkin lymphoma	site	9652/3	C81.2	9652/3
	Lymphocyte-depleted classic Hodgkin lymphoma	site	9653/3	C81.3	9653/3
	Classic Hodgkin lymphoma, NOS^c^	site	9650/3	C81.9	9650/3
*Follicular*	*In situ* follicular neoplasia		9695/1		
	Follicular lymphoma, grade 1	site	9695/3	C82.9	9690/3
	Follicular lymphoma, grade 2	site	9691/3	C82.9	9690/3
	Follicular lymphoma, grade 3	site	9698/3	C82.9	9690/3
	Follicular lymphoma, NOS	site	9690/3	C82.9	9690/3
	Primary cutaneous follicle centre lymphoma	C44	9597/3	C82.9	9690/3
*Diffuse large B-cell*	Diffuse large B-cell lymphoma (centroblastic, immunoblastic lymphoma, anaplastic)	site	9680/3	C83.8	9683/3
	Primary effusion lymphoma	site	9678/3	C83.8	9683/3
	Thymic large B-cell lymphoma	C37.9	9679/3	C83.8	9683/3
	Mediastinal large B-cell lymphoma	C38.3	9679/3	C83.8	9683/3
	T-cell/histiocyte-rich large B-cell lymphoma	site	9688/3	C83.8	9683/3
	Plasmablastic lymphoma	site	9735/3	C83.8	9683/3
	ALK-positive large B-cell lymphoma	site	9737/3	C83.8	9683/3
	Large B-cell lymphoma in HHV8-associated multicentric Castleman disease	site	9738/3	C83.8	9683/3
	B-cell lymphoma, unclassifiable, with features intermediate between diffuse large B-cell lymphoma and classic Hodgkin lymphoma	site	9596/3	C83.8	9683/3
	Intravascular large B-cell lymphoma	C49.9	9712/3	C84.4	9712/3
*Non-follicular*	Extranodal marginal zone lymphoma	site	9699/3	C82.9	9690/3
*indolent B-cell*	Nodal marginal zone lymphoma	C77	9699/3	C83.8	9711/3
	Splenic marginal zone lymphoma	C42.2	9689/3	C83.8	9711/3
	Other non-follicular lymphoma	site	9670/3	C83.8	9670/3
	Lymphoplasmacytic lymphoma	site	9671/3	C83.8	9671/3
	Splenic B-cell lymphoma, unclassifiable	C42.2	9591/3	C85.9	9591/3
*Mantle cell*	*In situ* mantle cell neoplasia		9673/1		
	Mantle cell lymphoma	site	9673/3	C83.8	9674/3
*Aggressive T-cell*	Blastic plasmacytoid dendritic cell neoplasm/blastic NK-cell lymphoma	site	9727/3	C83.8	9686/3
	Extranodal NK/T-cell lymphoma, nasal-type	site	9719/3	C84.4	9707/3
	Enteropathy-associated T-cell lymphoma	site	9717/3	C84.4	9707/3
	Hepatosplenic T-cell lymphoma	site	9716/3	C84.4	9707/3
	Angioimmunoblastic T-cell lymphoma	site	9705/3	C84.4	9705/3
	Subcutaneous panniculitis-like T-cell lymphoma	site	9708/3	C84.4	9707/3
	Peripheral T-cell lymphoma, NOS	site	9702/3	C84.4	9702/3
	Primary cutaneous gamma/delta T-cell lymphoma	C44	9726/3	C84.4	9702/3
	Anaplastic large cell lymphoma, ALK-positive	site	9714/3	C84.5	9714/3
	Anaplastic large cell lymphoma, ALK-negative	site	9715/3	C84.5	97143/5
	Early T-cell precursor lymphoblastic leukemia/lymphoma	site	9729/3	C84.5	96853/5
	Primary cutaneous anaplastic large cell lymphoma	C44	9718/3	C84.5	97143/5
	Lymphomatoid papulosis	C44	9718/3	C85.9	95903/5
*Other*	Precursor B-cell lymphoblastic lymphomas	site	9728/3	C83.5	96853/6
	Burkitt lymphoma	site	9687/3	C83.7	9687/3
	Blastic/aggressive non-Hodgkin lymphoma	site	95913/3	C83.8	9680/3
	Mycosis fungoides	C44	9700/3	C84.0	9700/3
	Sezary syndrome	C44	9701/3	C84.1	9701/3
	Cutaneous T-cell lymphoma	C44	9709/3	C84.4	9702/3
	Small cell/indolent non-Hodgkin lymphoma	site	95913/1	C85.0	9592/3
	B-cell lymphoma, NOS	site	95913/6	C85.1	95903/6
	Malignant lymphoma, suspected	site	95901/b	C85.9	95901/b
	Malignant lymphoma, NOS	site	9590/3	C85.9	9590/3
	Non-Hodgkin lymphoma, NOS	site	9591/3	C85.9	9591/3
	T-cell lymphoma, NOS	site	95913/5	C85.9	95903/5

a Diagnostic groups defined according to the National Quality Register for Lymphoma.^21^

b International Classification of Diseases for Oncology.

c NOS, Not otherwise specified.

All health-care providers are obliged under the Act on Health Data Registers (1998:543) and associated regulations to report all primary neoplasms to the National Cancer Register. The coverage of the register is close to 100% and 99% of the tumours are morphologically confirmed.^22^ To capture the age group that is most likely to be tattooed, we restricted inclusion to individuals aged 20–60 years at the time of diagnosis. Deceased cases were identified in the National Cause of Death Register and, if cases were deceased, next-of-kin was included to mitigate the impact of potential survivorship bias.

Three age- and sex-matched controls per case were randomly sampled from the population at risk on the date when the case was diagnosed (hereafter referred to as the index date). Controls were identified in the Total Population Register using incidence density sampling with person-time as the underlying timescale.

Individuals at risk of suffering psychological distress from study participation, according to diagnostic codes F20-29 in the International Statistical Classification of Diseases and Related Health Problems 10th Revision (ICD-10) in the National Patient Register, were not eligible as controls. Finally, indicator variables of socioeconomic status, i.e., educational attainment, disposable income of the household, and marital status, were added to the study population from the Longitudinal Integration Database for Health Insurance and Labor Market Studies, occupational history from the Swedish Occupational Register, and filled prescriptions from the National Prescribed Drug Register.

### Exposure assessment

We assessed exposure through a structured questionnaire administered by Statistics Sweden, the Swedish authority responsible for official statistics, in 2021. The data collection procedure has been described in detail elsewhere.^1^ Briefly, tattoos were defined as permanent designs received for decorative, cosmetic (defined as permanent make-up, semi-permanent makeup, and microblading), or medical (defined as skin restoration after mastectomy or burn lesions) purposes. Exposure was assessed as presence or absence of any tattoo, and respondents were explicitly asked to consider also removed tattoos. Respondents provided their age at first tattoo and tattoo characteristics including colours, total area of tattooed body surface (<1; 1–5; or >5 hand palms), skill level of tattooer (professional; amateur), and geographical region of tattooing. To mitigate the risk of selective participation, we informed participants that the purpose was to study new lifestyle factors in relation to cancer and other diseases, and included questions about several types of body modifications.

### Study size

We determined the sample size through an *a priori* power analysis. In the absence of previous studies, we based the power calculation on estimates from the epidemiologic literature on occupational exposures and lymphoma risk.^23^ We wanted to be able to detect an odds ratio of 1.3 with 80% power with a two-sided alpha level of 5%, assuming a tattoo prevalence of 17% among controls.^24^ This would be achieved by including 1300 cases and two controls per case. Acknowledging that national surveys have modest response rates (in 2021, the Swedish Public Health Survey and the SOM Institute's Public Opinion Survey had response rates of 44 and 50%, respectively),^25^^,^^26^ we scaled up the study population and included 3000 cases and three controls per case.

### Statistical analysis

In the study design phase, we used a directed acyclic graph (DAG) to visualize potential confounders of the association between tattoo exposure and malignant lymphoma. These assumptions formed the rational for the data collection strategy (Figure S1:1).

Statistics Sweden performed a drop-out analysis on aggregated data before data delivery. Individual-level data from non-responders was not available.

We used logistic regression to estimate the association between tattoo exposure and malignant lymphoma. In a case–control study with incidence-density sampling, the odds ratio provides an unbiased estimate of the incidence rate ratio (IRR) in the underlying population. We modelled exposure as a dichotomous variable (yes; no). To ascertain temporality between exposure and outcome, we required participants to have received their first tattoo before or at the index year to qualify as exposed. We addressed the impact of exposure duration by categorizing exposure according to the number of years between the first tattoo and the index year (0–2; 3–5; 6–10; or ≥11 years) and investigated a potential exposure-response relationship by categorizing the total area of tattooed body surface at the time of the survey. For the latter, we collapsed the higher exposure categories to reduce the risk of misclassification,^27^ and modelled the total tattooed body surface as <1 or >1 hand palm. Moreover, we assessed whether the colour scheme of the participants’ tattoos at the time of the survey (black/greys only; black/grey and colour) was associated with the risk of lymphoma. Finally, potential effect modification by laser treatment for tattoo removal was explored.

We used conditional logistic regression for the primary analyses to align with the study protocol. As balanced matching was performed (case:control ratio 1:3), we adjusted the models for the matching factors to control for both the original confounding and the selection bias introduced by the matching.^28^^,^^29^ Sex was entered as a dichotomous variable whereas age was modelled in five-year categories and with a term for residual age.^30^ In addition, we ran unconditional logistic regression models where the matched sets were broken to keep participants without a matching counterpart in the dataset and hence optimize precision.^29^ This approach has been shown to be valid for incidence-density matched data when the model adjusts for quintiles of time.^31^ We hereafter refer to these models as models with basic adjustments.

In fully adjusted models, we accounted for additional confounding by educational attainment (primary and lower secondary; upper secondary; or post-secondary), household disposable income (quartiles), self-reported smoking status (current; previous; or never smoker), and marital status (married or registered partnership; divorced or widowed; or unmarried) according to the DAG in Figure S1:2. Data on educational attainment and marital status applied to the index year, whereas household disposable income applied to the year before the index year to avoid differential misclassification from sickness benefit among cases. Household income was right-skewed, and we therefore categorized it according to the distribution among controls. The degree of missingness was very low (Table 2), and all analyses were therefore run on participants with complete sets of covariates.

**Table 2:** Sociodemographic characteristics and exposure status of the participating cases and controls.

	Cases (*n* = 1392)		Controls (*n* = 4179)	
	*n*	(%)^a^	*n*	(%)
Sex				
Male	769	55	2219	53
Female	623	45	1960	47
Age (years)^b^				
20–29	123	9	302	7
30–39	190	14	472	11
40–49	310	22	907	22
50–59	622	45	2006	48
≥60	147	11	492	12
Educational attainment^b^				
Primary/lower secondary	145	10	426	10
Upper secondary	616	44	1864	45
Post-secondary	629	45	1886	45
Missing	2	0.1	3	0.1
Marital status^b^				
Married/registered partnership	716	51	2182	52
Divorced/widowed	181	13	581	14
Unmarried	495	36	1416	34
Disposable income, household (SEK)^c^				
<310,900	411	30	1050	25
310,900–506,900	335	24	1043	25
507,000–696,200	335	24	1043	25
≥696,300	311	22	1043	25
Smoking status				
Current	134	10	470	11
Previous	467	34	1348	32
Never	785	56	2354	56
Missing	6	0.4	7	0.2
Tattoo status				
Yes	289	21	735	18
No	1097	79	3435	82
Missing	6	0.4	9	0.2

a Percentages not summing to 100 are caused by rounding.

b By December 31st in the index year.

c By December 31st in the year before the index year.

#### Subgroup analyses

We performed exploratory subgroup analyses to investigate the associations between tattoos and lymphoma subtypes, i.e., Hodgkin lymphoma, follicular lymphoma, diffuse large B-cell lymphoma, non-follicular indolent B-cell lymphoma (including marginal zone lymphoma), marginal zone lymphoma, mantle cell lymphoma, aggressive T-cell lymphoma, and other lymphomas. We modelled exposure as a dichotomous variable, and as a categorical variable accounting for exposure duration (0–2; 3–10; or ≥11 years).

#### Sensitivity analyses

We assessed the impact of confounding from having a) a hazardous occupation, or b) using immunosuppressive drugs. We hypothesised that both occupation, through workplace culture, and immunosuppressive therapy,^32^ through the underlying condition or through the drug *per se*, may affect an individual's inclination to get tattooed. Chemical exposures with sufficient or limited evidence for lymphoma were identified based on the International Agency for Research on Cancer's classification,^33^ (Table S1) and we considered individuals to have had a hazardous occupation if they, according to the Swedish Standard Classification of Occupations, had any of the listed occupations for at least one year before or at their index year. Likewise, we defined immunosuppressive therapy as ≥1 filled prescription with an Anatomical Therapeutic Chemical (ATC) code starting with L04A before the index date. The proportions that fulfilled these criteria were relatively low and we therefore assessed potential confounding through restriction.

We investigated potential survivorship bias by including the responses provided by deceased cases’ next-of-kin in the analysis.

It was standard procedure in Sweden to use permanent skin markings to guide patient positioning for radiotherapy throughout the study period. To evaluate potential reverse causation, we ran models where individuals who received their first tattoo within the same year as they were diagnosed with lymphoma were excluded.

The study was approved by the Swedish Ethical Review Authority (no. 2019-03138). Participants consented to participation by answering the questionnaire.

### Role of the funding source

The funding source had no role in the study design; in the collection, analysis, and interpretation of data; in the writing of the report; and in the decision to submit the paper for publication. All authors had full access to all data in the study and accept responsibility for the decision to submit for publication.

## Results

### Study participants

**Fig. 1:**
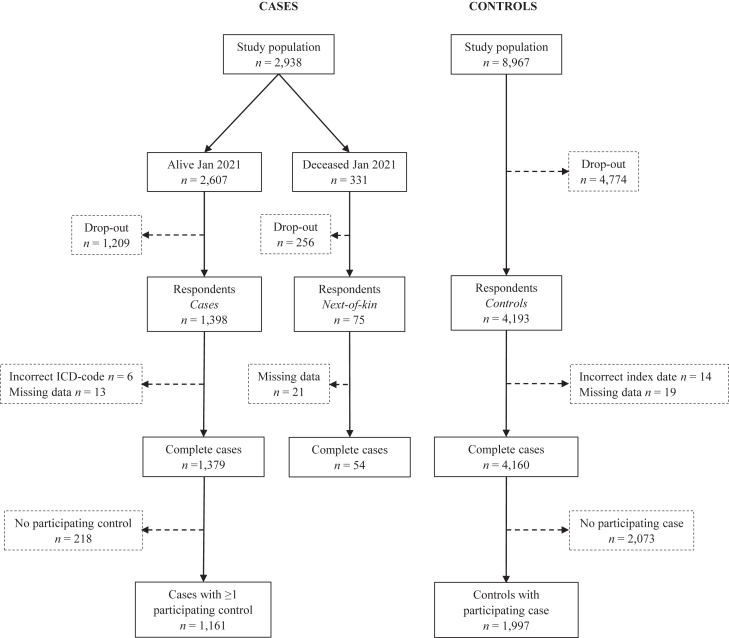
**Flowchart describing the inclusion of study participants**.

The study population consisted of 11,905 individuals (Fig. 1). At the time of the survey, 331 of the 2938 cases (11%) were deceased. The overall response rate was 48%, and the response rates among cases and controls were 54 and 47%, respectively.

The drop-out analysis showed that participants were older, more often born in Sweden, had higher educational attainment and household disposable income, and were more often married or in a registered partnership (Table S2). The proportion of males was higher among the nonparticipants.

The participating cases and controls were generally similar with respect to sociodemographic characteristics (Table 2). The tattoo prevalence was 21% among cases and 18% among controls. The degree of missingness in individual variables was very low (0.4% or less).

### Outcome and exposure characteristics

The most common subtypes were diffuse large B-cell lymphoma (28%), Hodgkin lymphoma (21%), and follicular lymphoma (18%) (Table 3). The median age at diagnosis ranged between 51 and 57 years, except for participants with Hodgkin lymphoma who were younger (median 36 years).

**Table 3:** Subtypes, sex distribution, and age at diagnosis of the 1392 participating cases with malignant lymphoma.

	*n*	(%)	Male (%)	Age at diagnosis (years) median (Q1; Q3)
Hodgkin	292	21	50	36 (28; 47)
Follicular	252	18	56	54 (47; 57)
Diffuse large B-cell	392	28	58	51 (44; 56)
Non-follicular indolent B-cell	136	10	49	55 (50; 58)
Marginal zone only	92		48	52 (43; 57)
Aggressive T-cell	61	4	67	52 (43; 58)
Mantle cell	61	4	66	57 (52; 59)
Other	198	14	55	52 (45; 58)

Tattooed cases and controls had received their first tattoo at approximately the same age (median ages 23 and 22 years, Table 4). Decorative tattoos were most common, but 7 and 8% of cases and controls, respectively, reported ever having a cosmetic tattoo. Compared with controls, a larger proportion of cases had received a medical tattoo. None of the variables assessing the total area of tattooed body surface indicated a difference between cases and controls. Likewise, the colour schemes were comparable. Most individuals got tattooed by a professional tattooer, and the proportions that were tattooed by a nonprofessional were 14% among cases and 13% among controls. A slightly lower proportion of cases than controls were tattooed in Sweden, whereas a higher proportion of cases had been tattooed in Asia. Finally, although numbers were small, a larger proportion of cases had undergone laser treatment for tattoo removal.

**Table 4:** Exposure characteristics among the cases and controls that received their first tattoo before or during the index year. Results are displayed as median (Q1; Q3) or *n* (%).

	Cases (*n* = 289)		Controls (*n* = 735)	
	*n*	Median (Q1; Q3)	*n*	Median (Q1; Q3)
Age at first tattoo, years	289	23 (19; 35)	735	22 (18; 35)
Years of exposure at index year	289	15 (7; 26)	735	17 (9; 27)
	*n*	(%)^a^	*n*	(%)
Tattoo type^b^				
Decorative	267	92	705	96
Cosmetic	19	7	58	8
Medical	25	9	14	2
Area of tattooed body surface				
<1 hand	159	55	372	51
1–5 hands	101	35	259	35
>5 hands	27	9	97	13
Missing	2	1	7	1
Number of tattoos^c^				
1	115	40	313	43
2–3	99	34	216	29
4–5	38	13	88	12
6–9	20	7	70	10
≥10	14	5	44	6
Missing	3	1	4	1
Number of tattoo sessions				
1	125	43	311	42
2–3	93	32	212	29
4–5	31	11	89	12
6–9	17	6	60	8
≥10	19	7	56	8
Missing	4	1	7	1
Colour scheme				
Only black/grey	113	39	254	35
Only colour	42	15	116	16
Combination of black/grey and colour	133	46	362	49
Missing	4	1	4	1
Ink colours^b^				
Black	245	85	608	83
Grey	36	12	132	18
Brown	27	9	58	8
Red	96	35	294	41
Blue	88	33	240	33
Green	82	28	236	32
Yellow	59	20	196	27
White	46	16	129	18
Purple	12	4	56	8
Pink	9	3	61	8
Orange	21	7	53	7
Turquoise	17	6	54	7
Skin tone (injected colour)	3	1	7	1
Other	6	2	4	1
Tattooer and location^b^				
Professional, in studio	230	80	620	84
Professional, other facility	39	13	101	14
Cosmetic tattooer, in studio or clinic	11	4	44	6
Healthcare professional, in hospital	25	9	11	1
Other, irrespective of where	41	14	95	13
Geographical regions of tattooing^b^				
Sweden	237	82	646	88
Nordic countries (not Sweden)	21	7	58	8
Rest of Europe (not Nordic countries)	32	11	76	10
Asia	32	11	52	7
Oceania	3	1	11	1
USA	9	3	23	3
Other	8	3	15	2
Laser removal of tattoo				
Yes	13	5	13	2
No	275	95	712	97
Missing	1	0.4	10	1

a For single-answer questions, proportions not summing to 100 are caused by rounding.

b Multiple answers were possible. Proportions are calculated with the total number of tattooed cases or controls in the denominator.

c Tattoos considered separate entities when ≥20 cm apart.

### Association between tattoo exposure and lymphoma

In the matched analysis, tattooed participants had a higher adjusted risk of malignant lymphoma than nontattooed participants (IRR = 1.21; 95% CI 0.99–1.48) (Table 5). When the matches were broken, the estimate was slightly attenuated but more precise (IRR = 1.18, 95% CI 1.01–1.39).

**Table 5:** Incidence rate ratios (IRR) of malignant lymphoma in tattooed individuals from unmatched and matched analyses with different approaches to exposure assessment.

Exposure assessment	Matched analysis								Unmatched analysis							
	Basic adjustment^a^				Full adjustment^b^				Basic adjustment^c^				Full adjustment^d^			
	Cases (*n*)	Controls (*n*)	IRR^e^ (95% CI)	*p*-value	Cases (*n*)	Controls (*n*)	IRR (95% CI)	*p*-value	Cases (*n*)	Controls (*n*)	IRR (95% CI)	*p*-value	Cases (*n*)	Controls (*n*)	IRR (95% CI)	*p*-value
*Tattoo status (index year)*				*0.030*				*0.067*				*0.039*				*0.040*
Tattooed	241	334	1.24 (1.02–1.50)		241	333	1.21 (0.99–1.48)		289	735	1.18 (1.01–1.38)		289	733	1.18 (1.01–1.39)	
Nontattooed	927	1668			920	1664			1097	3435	1.00		1090	3427	1.00	
*Exposure duration (years between first tattoo and index year)*				*0.12*				*0.19*				*0.034*				*0.039*
0–2	28	26	1.83 (1.05–3.21)		28	26	1.81 (1.03–3.20)		33	50	1.96 (1.25–3.08)		33	50	1.96 (1.25–3.09)	
3–5	15	25	1.01 (0.52–1.98)		15	24	0.97 (0.49–1.95)		21	57	1.05 (0.62–1.76)		21	56	1.07 (0.63–1.80)	
6–10	33	52	1.12 (0.71–1.77)		33	52	1.11 (0.70–1.77)		47	128	1.06 (0.75–1.50)		47	127	1.06 (0.74–1.51)	
≥11	165	231	1.23 (0.98–1.53)		165	231	1.19 (0.94–1.50)		188	500	1.15 (0.96–1.38)		188	500	1.15 (0.95–1.39)	
Nontattooed	927	1668	1.00		920	1664	1.00		1097	3435	1.00		1090	3427	1.00	
*Tattooed body surface (at the time of the survey)*				*0.075*				*0.14*				*0.022*				*0.022*
<1 hand palm	139	188	1.29 (1.01–1.65)		139	188	1.27 (0.99–1.63)		159	372	1.33 (1.09–1.62)		159	372	1.33 (1.09–1.63)	
>1 hand palm	100	142	1.18 (0.90–1.56)		100	141	1.14 (0.86–1.53)		128	356	1.03 (0.83–1.28)		128	355	1.03 (0.82–1.29)	
Nontattooed	927	1668	1.00		920	1664	1.00		1097	3435	1.00		1090	3427	1.00	
*Colour scheme (at the time of the survey)*				*0.081*				*0.16*				*0.063*				*0.061*
Black/grey	86	114	1.24 (0.91–1.68)		86	113	1.23 (0.91–1.68)		113	253	1.31 (1.03–1.66)		113	252	1.32 (1.04–1.68)	
Black/grey and colour	154	217	1.25 (1.00–1.57)		154	217	1.21 (0.95–1.54)		175	479	1.11 (0.92–1.34)		175	478	1.11 (0.92–1.35)	
Nontattooed	927	1668	1.00		920	1664	1.00		1097	3435	1.00		1090	3427	1.00	
*Effect modification, laser treatment (before the survey)*				*0.026*				*0.051*				*0.006*				*0.006*
Tattooed, without laser treatment	230	323	1.22 (1.01–1.49)		230	322	1.19 (0.97–1.46)		275	712	1.16 (0.99–1.36)		275	711	1.17 (0.99–1.37)	
Tattooed, with laser treatment	10	7	2.66 (0.99–7.17)		10	7	2.63 (0.96–7.18)		13	13	2.93 (1.35–6.36)		13	13	2.99 (1.37–6.52)	
Nontattooed	927	1668	1.00		920	1664	1.00		1097	3435	1.00		1090	3427	1.00	

a Estimates obtained from conditional logistic regression models adjusted for sex and age.

b Estimates obtained from conditional logistic regression models adjusted for sex, age, educational attainment, smoking, marital status, and household disposable income.

c Estimates obtained from unconditional logistic regression models adjusted for sex, age, and index year.

d Estimates obtained from unconditional logistic regression models adjusted for sex, age, index year, educational attainment, smoking, marital status, and household disposable income.

e Incidence rate ratio.

The risk of lymphoma was highest in individuals with less than two years between their first tattoo and the index year (IRR = 1.81; 95% CI 1.03–3.20). The risk decreased with intermediate exposure duration (i.e., three to ten years), but seemed to increase again in individuals who received their first tattoo ≥11 years before the index year (IRR = 1.19; 95% CI 0.94–1.50). We found no evidence of an increased risk with a larger total area of tattooed body surface. On the contrary, we observed the highest lymphoma risk in individuals with tattoos smaller than one hand palm. The estimates for different colour schemes were similar in the matched analysis (IRR = 1.23; 95% CI 0.91–1.68 for black/grey tattoos only, and IRR = 1.21; 95% CI 0.95–1.54 for black and coloured tattoos), but the unmatched analysis was suggestive of a higher risk associated with having black/grey tattoos only (IRR:1.32; 95% CI 1.04–1.68) compared with having both black and coloured tattoos (IRR = 1.11; 95% CI 0.92–1.35).

Laser treatment for tattoo removal seemed to drastically modify the risk of lymphoma. Among tattooed participants who had undergone laser treatment, the relative risk of lymphoma in the matched analysis was 2.63 (95% CI 0.96–7.18), although the estimate was associated with a wide confidence interval because of small numbers. When we retained more data and performed an unmatched analysis, the estimate indicated substantial effect modification by tattoo laser removal (IRR = 2.99; 95% CI 1.37–6.52).

#### Subgroup analyses

**Fig. 2:**
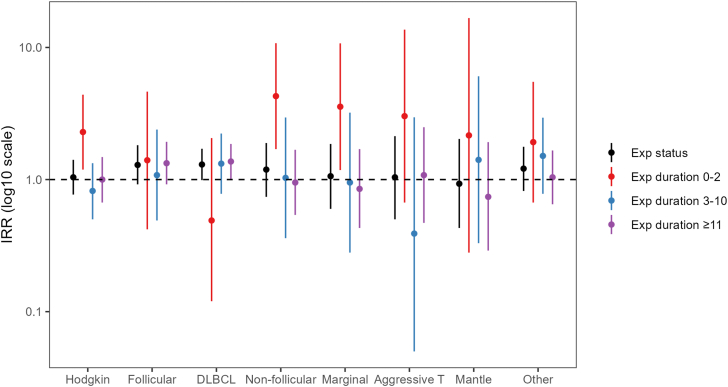
**Adjusted incidence rate ratios (IRR) of malignant lymphoma subtypes in tattooed relative to nontattooed participants. The error bars represent the upper and lower limits of the 95th confidence intervals of the estimates. Tattoo exposure was modelled as a) exposure status (yes; no), and b) exposure duration (years). The underlying numeric data for this figure are presented in**Table S3**. DLBCL, diffuse large B-cell lymphoma**.

The risk associated with tattoo exposure was strongest for diffuse large B-cell lymphoma (IRR 1.30; 95% CI 0.99–1.71), followed by follicular lymphoma (IRR 1.29; 95% CI 0.92–1.82) (Fig. 2; Table S3). When we accounted for exposure duration, the risk for diffuse large B-cell lymphoma seemed to be highest in participants with ≥11 years between their first tattoo and the index year, whereas the risk for follicular lymphoma seemed to be elevated both in individuals with 0–2 years between their first tattoo and the index year and with ≥11 years between the first tattoo and the index year. The results were suggestive of an increased risk in individuals with <2 years between their first tattoo and the index year for both Hodgkin lymphoma and non-follicular indolent B-cell lymphoma, but we observed no effect at longer exposure durations.

#### Sensitivity analyses

The proportions of participants with a hazardous occupation at or before their index year were comparable between cases (*n* = 63; 5%) and controls (*n* = 200; 5%). The results did not change markedly when we restricted the analysis to individuals that had never held a hazardous occupation (Table 6).

**Table 6:** Sensitivity analyses evaluating the impact of various analytical decisions on the effect estimates.

	Basic adjustment^a^				Full adjustment^b^			
	Cases (*n*)	Controls (*n*)	IRR^c^ (95% CI)	*p*-value	Cases (*n*)	Controls (*n*)	IRR (95% CI)	*p*-value
*Restriction: No hazardous occupation (at or before index year)*				*0.071*				*0.13*
Tattooed	228	315	1.20 (0.99–1.47)		228	314	1.18 (0.96–1.45)	
Nontattooed	890	1591	1.00		883	1587	1.00	
*Restriction: No immunosuppressive drugs (at or before index year)*				*0.029*				*0.049*
Tattooed	229	326	1.25 (1.02–1.52)		229	325	1.23 (1.00–1.52)	
Nontattooed	872	1643	1.00		866	1639	1.00	
*Inclusion: Next-of-kin*				*0.039*				*0.078*
Tattooed	251	352	1.22 (1.01–1.47)		249	351	1.20 (0.98–1.46)	
Nontattooed	982	1763	1.00		960	1759	1.00	
*Restriction: No (first) tattoo in index year*				*0.048*				*0.089*
Tattooed	232	326	1.22 (1.00–1.48)		232	325	1.19 (0.97–1.46)	
Nontattooed	936	1676	1.00		929	1672	1.00	

a Estimates obtained from conditional logistic regression models adjusted for sex and age.

b Estimates obtained from conditional logistic regression models adjusted for sex, age, educational attainment, smoking, marital status, and household disposable income.

c Incidence rate ratio.

Immunosuppressive therapy was more common among cases, where 6% (*n* = 78) used immunosuppressant drugs at or before the index year compared with 2% (*n* = 75) among controls. Restricting the analysis to individuals who had not received immunosuppressive therapy resulted in a slight amplification of the effect estimate.

Next-of-kin reported a tattoo prevalence in deceased cases of 19% (*n* = 14). The results did not change when (the few) next-of-kin responses were included in the analysis. Likewise, the estimates remained the same when we restricted the dataset to participants who had received their first tattoo before the index year.

## Discussion

There is an ongoing discourse regarding whether or not tattoos are linked to malignancies. More than a decade ago, a literature review compared the number of published case reports of (cutaneous) malignancies in tattoos with the size of the global tattooed population, and concluded that an association between tattoo exposure and (skin) cancers was likely to be coincidental.^34^ Although case reports are useful to highlight new research questions and generate hypotheses for future research, they suffer from inherent limitations that hinder causal inference, such as selective reporting and the lack of comparison groups. In the absence of well-designed epidemiologic research, there has been no scientific basis for a sound evaluation of the potential link between tattoo exposure and cancer.

Our results suggest that tattooed individuals have a 21% increased risk of overall lymphoma relative to nontattooed individuals. To our knowledge, the only published epidemiologic study of tattoo exposure and lymphoma is the work by Warner and coworkers,^20^ but their investigation was likely underpowered because of the low number of tattooed participants. Thus, our study is the first to provide insight into a potential association between tattoo exposure and lymphoma using a population-based study design with a large sample size. However, causality cannot be conferred from a single epidemiologic study, and more research is needed.

Carcinogenesis is a multistage process of cellular transformation, simplistically described in terms of initiation, promotion, and progression. Exogenous chemical carcinogens can act at one or several stages of this process, often in complex interaction with other agents.^35^ Optimally, understanding of an agent's biologic mode of action should inform the analytical strategy with respect to exposure latency in epidemiologic studies. Because tattoo inks are heterogeneous chemical mixtures, this was not possible in the current study. Instead, we explored the impact of exposure duration by accounting for the number of years between the first tattoo and the index year. The results suggested that both tattoos received 0–2 years before the index year and tattoos received ≥11 years before the index year may be associated with an increased lymphoma risk. If these findings can be corroborated by further studies, they would indicate that exposure to tattoo ink may be associated with both tumour initiation, which is often associated with a latency of several years, and tumour promotion where effects occur much faster. A time-dependent effect seems plausible considering the toxicokinetics of tattoo ink because the chemical composition of the exposure is likely to be dynamic. Shortly after tattooing, the exposure mainly consists of solid-state material (i.e., pigment particles), substances attached to the pigment particles (e.g., PAH and PAA), and various soluble compounds. It can be assumed that the solid-state material is deposited in the lymph nodes, whereas soluble substances are metabolized faster. However, the toxicokinetics of tattoo ink is an under-researched area that warrants attention to improve current risk assessments.

On the contrary to what we expected, we observed the greatest risk associated with (first) tattoos received less than two years before the index year. We suspected that this finding could be the results of reverse causation because the prevalence of medical tattoos was higher in cases (9% compared with 2% in controls), and application of permanent skin markings to guide patient positioning for radiotherapy was standard procedure during the study period. However, the estimate did not change when we excluded individuals who received their first tattoo during the index year, which suggests that there could be more to the risk associated with recent tattoos than bias.

It seems intuitive that a larger tattooed body surface would infer a greater health risk than a small tattoo, but we did not find evidence of an exposure-response relationship even though we collapsed the higher exposure categories to reduce the risk of exposure misclassification. Likewise, we did not observe a distinct difference in risk associated with the different colour schemes despite the distinct differences in the chemical composition of black and coloured inks. The area of tattooed body surface and the tattoo colour scheme were assessed at the time of the survey, whereas exposure status (with respect to the first tattoo) was assessed in the index year. In participants with more than one tattoo, the current data may not represent the tattooed body surface and/or the colour scheme in the index year, resulting in misclassification. Finally, the study was not designed to investigate an exposure-response relationship, nor to distinguish between colour schemes. These analyses were likely underpowered and should be interpreted as exploratory.

An intriguing finding was that laser treatment for tattoo removal modified the effect of exposure and resulted in a substantially higher risk estimate. These results align with evidence from experimental studies demonstrating cleavage of azo compounds in tattoo ink into carcinogenic aromatic amines like o-toluidine, 2-amino-4-nitrotoluene and 3,3′-dichlorobenzidine toxic compounds after laser irradiation.^36^ Clearly, not only the long-term health effects of tattoo exposure *per se* but also the implications of laser treatment for tattoo removal warrants further investigation in light of the potential public health implications.

The results were suggestive of an increased risk of B-cell lymphomas, particularly diffuse large B-cell lymphoma and follicular lymphoma. In contrast, Warner et al. observed a tendency towards an increased risk of T-cell lymphoma, although the overall result of their study was null. However, neither of the studies was powered to investigate lymphoma subtypes, and the estimates were associated with wide confidence intervals.

It seems reasonable that immune disruption caused by tattoo-related chemicals deposited within the lymphatic system may explain a potential association between tattoo exposure and lymphoma. Exposure to some PAH is associated with reduced immune surveillance or immunosuppression of cancer cells.^37^ Several autoimmune disorders are established risk factors for diffuse large B-cell lymphoma,^38^ and exogenous exposures (i.e., implants) have been described in case reports of patients with diffuse large B-cell lymphoma.^39^, ^40^, ^41^ The research area would benefit from an increased understanding of the pathobiological mechanisms that may link tattoo exposure to cancer.

Our study has several strengths. Firstly, it is the first epidemiologic study to investigate the association between tattoo exposure and malignant lymphoma using a population-based design and a large sample size. Secondly, we undertook several measures to limit the impact of potential bias.

We included all incident cases diagnosed in Sweden during the study period, which reduces the risk of outcome misclassification to a minimum. Because Swedish National Registers have full population coverage and because this case–control study was nested within the Total Population Register, the controls were truly representative of the underlying population that generated the cases (i.e., the total Swedish population). The full population coverage applied also to data on potential confounders, which resulted in a neglectable degree of missingness.

We consider recall bias with respect to exposure *status* to be highly unlikely because getting a tattoo is an active decision underpinned by motivational factors such as achievement of milestones or expression of individuality or affection.^42^ In addition, we do not expect individuals with lymphoma to systematically misremember getting tattooed to a higher (or lower) extent than individuals without lymphoma, which would be the definition of recall bias within the current study design.

To accurately define an appropriate latency period between exposure and outcome poses an inherent challenge in investigations of understudied exposures, particularly so when the exposure is a heterogeneous mixture of chemicals. To overcome this uncertainty, we ran models with exposure categorized according to the number of years that had elapsed between the first tattoo and the index year. The validity of self-reported tattoo exposure *duration* (i.e., age at first tattoo) has to the best of our knowledge never been studied. However, because of the explicit motivational factors of tattooing, we expect that participants remembered their age when they got tattooed for the first time, and hence consider the risk of recall bias with respect to exposure duration a minor concern.

Although our assessment of total tattooed body surface showed strong inter-rater agreement in a pilot study, Foerster et al. found that study participants tended to overestimate their tattooed body surface.^27^ It seems reasonable to assume that the risk of exposure misclassification would be the same in cases and controls, and hence non-differential. However, if there is a true exposure–response relationship and participants would tend to overestimate their tattooed body surface, then the effect estimate of the higher-exposure category would be biased towards the null. The lack of data on tattoo coverage (the inked area of the total tattoo area) could possibly act to superimpose the exposure misclassification if smaller tattoos would contain a higher quantity of ink per unit of tattooed skin than larger tattoos. These mechanisms may partly explain the absence of a monotonous exposure-response relationship, but they do not explain why the estimate of the higher exposure category was lower than that of the lower exposure category. However, a limiting factor in this analysis was clearly that the assessment of tattooed body area was performed at the time of the survey, and not with respect to the index year. Future studies can overcome this limitation by asking study participants to report all the years they received tattoos. In addition, it would be useful to ask about the type of tattoo (i.e., decorative, cosmetic, and medical) received at each point in time as tattoo type may modify the association with lymphoma. The latter was not possible to address in the current study.

A limitation was that there were differences between participants and nonparticipants in terms of sociodemographic characteristics. However, we have no reason to believe that the *association* between exposure and outcome would differ depending on these variables to such an extent that it would hamper the generalizability. Caution should however be taken in generalizing the results to individuals older than 60 years because the aetiology of lymphoma may differ depending on age at diagnosis. Selective participation is a generic concern that may distort the results of any case–control study. The fact that we could not formally assess potential selection bias because we did not have access to individual-level data on the nonparticipants is a limitation. Nevertheless, we consider the risk of selection bias to be limited because of precautionary measures in the study design (i.e., it was not evident for the participants that the purpose was to study the association between tattoo exposure and cancer), similar response rates in cases and controls, and a tattoo prevalence that was comparable to that of the Swedish National Environmental Health Survey from 2015 (considering that our data collection took place six years later).^24^

We could not fully rule out survivorship bias because of the low response rate among deceased cases' next-of-kin. Hence, if tattoo exposure is associated with increased mortality in patients with lymphoma, we might have underestimated the true risk. However, 89% of the cases were still alive at the time of the survey and the tattoo prevalence reported by deceased cases’ next-of-kin was comparable to that of the participating cases. We therefore consider the risk of survivorship bias to be low.

Finally, although the models were adjusted for important confounders such as educational attainment and income, we cannot rule out the risk of residual confounding from unmeasured confounders or measurement error in self-reported confounders (i.e., smoking).

Our results suggest that tattooed individuals have an increased risk of lymphoma which underscores the need for continued research into the long-term health effects of tattoos. Causality cannot be conferred from a single epidemiologic study and the results need to be confirmed by further research.

## Contributors

Christel Nielsen: Conceptualization; methodology; software; formal analysis; investigation; resources; data curation; writing–original draft; visualization; project administration; and funding acquisition, Mats Jerkeman: Writing–review & editing, Anna Saxne Jöud: Methodology; validation of underlying data; and writing–review & editing.

All authors had full access to all the data in the study and accept responsibility for the decision to submit for publication.

## Data sharing statement

The data collected for the study contain sensitive personal information that cannot be publicly shared. Data can be made available for collaborative research projects approved by the Swedish Ethical Review Authority. Any data inquiries should be addressed to the corresponding author.

## Declaration of interests

The authors declare no conflicts of interest.
