# Supplementary material for: Tattoos as a risk factor for malignant lymphoma: a population-based case–control study
Source: eClinicalMedicine. 2024 May 21;72:102649. doi: 10.1016/j.eclinm.2024.102649 (PMC11141277; doi:10.1016/j.eclinm.2024.102649)
Supplement: Supplementary Figures and Tables [file mmc1.pdf]

Supplementary

Table of contents

Figure S1. The directed acyclic graph used to visualize the underlying associations between variables related to tattoo exposure and/or malignant lymphoma during the design phase of the study. 2

Figure S2. The directed acyclic graph used to inform the statistical analyses. 3

Table S1. Occupations with potential exposure to chemicals with sufficient or limited evidence for lymphoma according to the International Agency for Research on Cancer's classification. 4

Table S2. Descriptive drop-out analysis of the 5,591 responders and the 5,983 non-responders at the time of the survey stratified on outcome status. 5

Table S3. Subgroup analyses of the association between a) tattoo exposure status, and b) tattoo exposure duration, and malignant lymphoma subtypes. 6

References 7

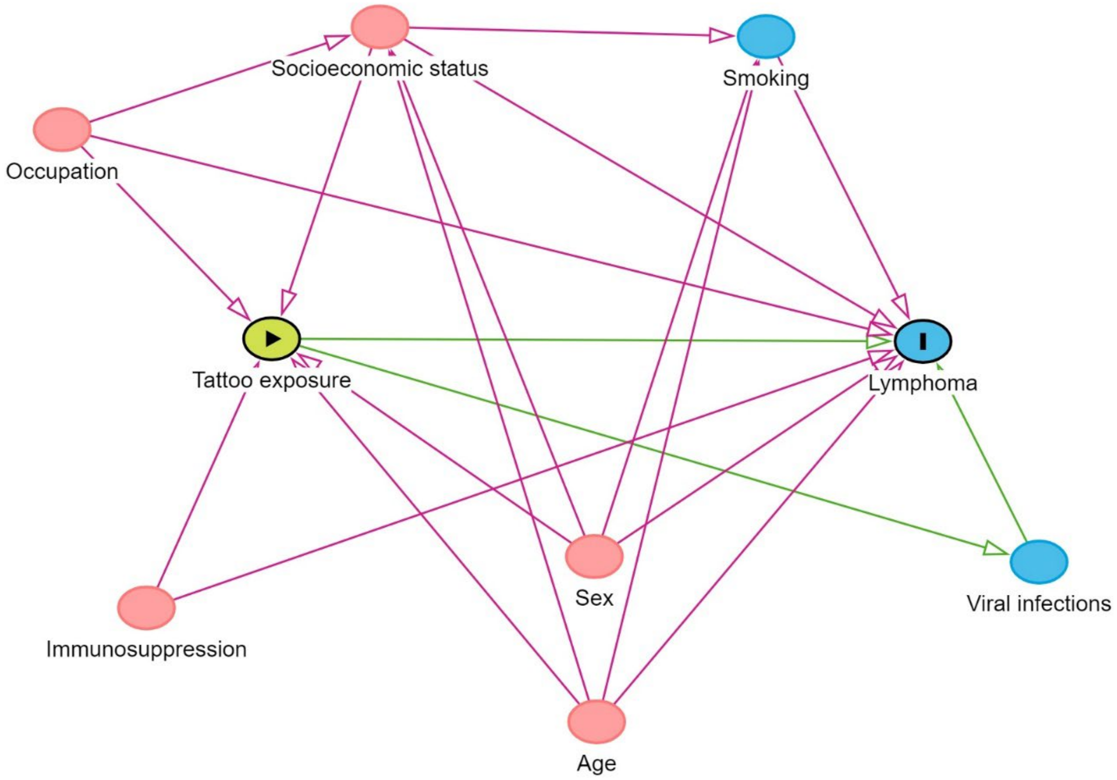

Figure S1. The directed acyclic graph used to visualize the underlying associations between variables related to tattoo exposure and/or malignant lymphoma in the design phase. [1] We used register data on educational attainment, household disposable income, and marital status as proxy variables for socioeconomic status. Viral infections constitute a mediator that should not be adjusted for.

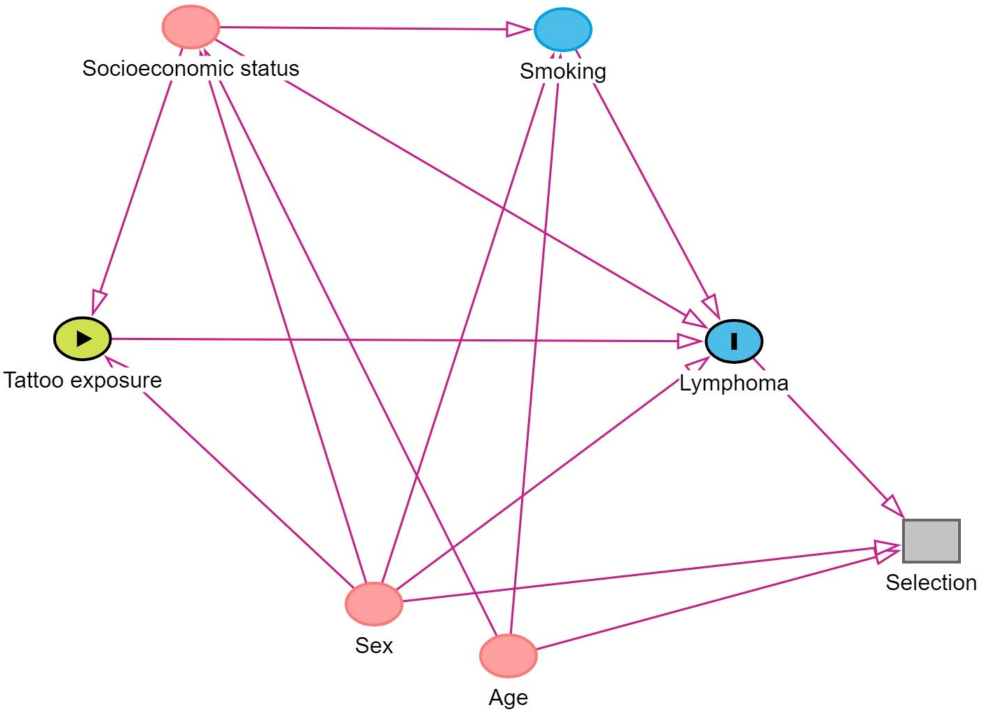

Figure S2. The directed acyclic graph after case-control matching on age and sex. The green arrow represents the association under study. To correctly account for both confounding and the selection bias introduced by the matching, models should adjust for age, sex, socioeconomic status, and smoking. [2]

Table S1. Occupations with potential exposure to chemicals with sufficient or limited evidence for lymphoma according to the International Agency for Research on Cancer's classification. [3]

	SSYK[1] 2012	SSYK 96
Field crop and vegetable growers	6111	1311, 6111
Gardeners, cultivation	6112	1311, 6112
Berry pickers and planters	9210	9210
Wood treaters	7521	7421
Machine operators, rubber products	8141	8231
Machine operators, plastic products	8142	8232
Machine operators, wood treaters	8174	8240
Chemical processing plant controllers	8192	8150
Metal-, rubber-, and plastic-products assemblers	8213	8283, 8290
Firefighters	5411	5151
Hairdressers	5141	5141, 5142, 5143, 5144, 5149, 5419

[1]SSYK = Swedish Standard Classification of Occupations.

Table S2. Descriptive drop-out analysis of the 5591 participants and the 5983 nonparticipants at the time of the survey.

	Cases (*n*=2607)				Controls (*n*=8967)			
	Participants (*n*=1398)		Nonparticipants (*n*=1209)		Participants (*n*=4193)		Nonparticipants (*n*=4774)	
	n	(%)[1]	*n*	(%)	*n*	(%)	*n*	(%)
Sex								
Male	772	55	724	60	2226	53	2982	63
Female	626	45	485	40	1967	47	1792	38
Age								
20-29	39	3	78	7	97	2	273	6
30-39	142	10	213	18	384	9	738	16
40-49	240	17	244	20	566	14	1012	21
50-59	421	30	366	30	1284	31	1385	29
60-69	554	40	308	26	1859	44	1363	29
70-79	2	0.1	-	-	3	0.1	3	0.1
Educational attainment								
Primary and lower secondary	126	9	207	17	366	9	758	16
Upper secondary	596	43	617	51	1826	44	2433	51
Post-secondary	673	48	377	31	1996	48	1512	32
Missing	2	0.1	8	0.8	3	0.1	67	1
Country of birth								
Sweden	1189	85	830	69	3680	88	3500	73
Other	209	15	379	31	513	12	1274	27
Citizenship								
Swedish	1364	98	1118	93	4095	98	4420	93
Other	34	2	91	8	98	2	354	7
Marital status								
Married	755	54	495	41	2300	55	2014	42
Unmarried	428	31	474	39	1237	30	1872	39
Divorced	197	14	232	19	594	14	816	17
Widowed	18	1	8	1	62	2	72	2
Disposable income (SEK)								
None (0)	26	2	83	7	69	2	247	5
1 – 124,999	98	7	136	11	213	5	471	10
125,000 – 199,999	94	7	123	10	271	7	426	9
200,000 – 279,999	163	12	165	14	462	11	615	13
280,000 – 369,999	305	22	275	23	849	20	1122	24
≥370,000	712	51	427	35	2329	56	1893	40

[1]Percentages not summing to 100 are caused by rounding.

Table S3. Exploratory subgroup analyses of the association between a) tattoo exposure status, and b) tattoo exposure duration, and malignant lymphoma subtypes.

	Exposure status	Cases (*n*)	Controls (*n*)	Adjusted IRR[1] (95 % CI)	*p*-value	Exposure duration (years)	Cases (*n*)	Controls (*n*)	Adjusted IRR (95 % CI)	*p*-value
Hodgkin	Tattooed	77	733	1.04 (0.77-1.41)	0.80	0-2	15	50	2.29 (1.19-4.39)	0.058
						3-10	25	183	0.82 (0.50-1.33)	
						≥11	37	500	1.00 (0.67-1.48)	
	Nontattooed	212	3427	1.00		Nontattooed	212	3427	1.00	
Follicular	Tattooed	49	733	1.29 (0.92-1.82)	0.14	0-2	3	50	1.40 (0.42-4.63)	0.48
						3-10	7	183	1.08 (0.49-2.39)	
						≥11	39	500	1.33 (0.92-1.93)	
	Nontattooed	199	3427	1.00		Nontattooed	199	3427	1.00	
Diffuse large B-cell	Tattooed	82	733	1.30 (0.99-1.71)	0.057	0-2	2	50	0.49 (0.12-2.06)	0.11
						3-10	18	183	1.32 (0.78-2.23)	
						≥11	62	500	1.37 (1.01-1.86)	
	Nontattooed	307	3427	1.00		Nontattooed	307	3427	1.00	
Non-follicular indolent B-cell	Tattooed	25	733	1.19 (0.74-1.89)	0.47	0-2	6	50	4.28 (1.70-10.77)	0.021
						3-10	4	183	1.03 (0.36-2.95)	
						≥11	15	500	0.95 (0.54-1.68)	
	Nontattooed	109	3427	1.00		Nontattooed	109	3427	1.00	
Marginal zone only	*Tattooed*	*17*	*733*	*1.06 (0.60-1.86)*	*0.85*	*0-2*	*4*	*50*	*3.56 (1.18-10.74)*	*0.13*
						3-10	*3*	*183*	*0.95 (0.28-3.21)*	
						≥11	*10*	*500*	*0.85 (0.43-1.70)*	
	Nontattooed	*73*	*3427*	*1.00*		*Nontattooed*	*73*	*3427*	*1.00*	
Aggressive T-cell	Tattooed	10	733	1.04 (0.50-2.13)	0.93	0-2	2	50	3.02 (0.67-13.64)	0.37
						3-10	1	183	0.39 (0.05-2.96)	
						≥11	7	500	1.08 (0.47-2.49)	
	Nontattooed	51	3427	1.00		Nontattooed	51	3427	1.00	
Mantle cell	Tattooed	8	733	0.93 (0.43-2.03)	0.86	0-2	1	50	2.16 (0.28-16.69)	0.75
						3-10	2	183	1.41 (0.33-6.05)	
						≥11	5	500	0.74 (0.29-1.92)	
	Nontattooed	53	3427	1.00		Nontattooed	53	3427	1.00	
Other	Tattooed	38	733	1.21 (0.82-1.77)	0.34	0-2	4	50	1.92 (0.67-5.49)	0.43
						3-10	11	183	1.51 (0.78-2.94)	
						≥11	23	500	1.04 (0.65-1.66)	
	Nontattooed	159	3427	1.00		Nontattooed	159	3427	1.00	

All estimates were obtained from unconditional logistic regression adjusted for age, sex, index year, educational attainment, household disposable income, marital status, and smoking. There were too few observations to allow for matched analyses.

[1]Incidence rate ratio.

References

1. Textor J, van der Zander B, Gilthorpe MS, Liśkiewicz M, Ellison GT. Robust causal inference using directed acyclic graphs: the R package ‘dagitty’. *Int J Epidemiol* 2017; 45(6): 1887-94.
2. Shrier I, Stovitz SD, Textor J. Identifiability of causal effects in test-negative design studies. *Int J Epidemiol* 2023; 52(6): 1968-74.
3. World Health Organization, International Agency for Research on Cancer. List of classifications by cancer sites with *sufficient* or *limited evidence* in humans. 2023: IARC Monographs. https://monographs.iarc.who.int/wp-content/uploads/2019/07/ Classifications_by_cancer_site.pdf. Accessed 2024-03-10
